# Supplementary material for: Cytotaxonomy and Molecular Analyses of Mycteria americana (Ciconiidae: Ciconiiformes): Insights on Stork Phylogeny
Source: Genes (Basel). 2023 Mar 28;14(4):816. doi: 10.3390/genes14040816 (PMC10138051; doi:10.3390/genes14040816)
Supplement: Supplementary file 1 [file genes-14-00816-s001.zip › genes-2280980-SI.pdf]

Table S1. Data on the species analyzed in this study, their respective GenBank and/or Boldsystems accession codes.

Species	Cytochrome oxidase I (COI)		Cytochrome b (Cytb)
	Genbank	Boldsystems	
Anastomus oscitans	KY040008.1	-	-
	KY040009.1	-	-
Ciconia abdimii	KU722448.1	GBIR7208-17	U72774.1
	MT456662.1	SIBSI057-19	-
	-	ROMC112-07	-
Ciconia boyciana	KJ862146.1	GBIR9434-19	AB026193.1
	KJ862145.1	GBMIN133497-17	NC_002196.1
	KJ862153.1	GBMIN133504-17	U72773.1
Ciconia ciconia	KY754495.1	MPBM010-17	AB026818.1
	NC_002197.1	GBMTG125-16	NC_002197.1
	MT773178.1	GBMNC107-20	MN122897.1
	AB026818.1	GBMNA11117-19	KJ456229.1
	AY567881.1	GBIR1245-09	U70822.1
	GU571816.1	BISE414-08	X86744.1
	GU571817.1	BISE237-08	AY567909.1
	MZ050101.1	-	-
	MZ050102.1	-	-
	MZ050103.1	-	-
Ciconia episcopus	U72775.1	-	-
	U72785.1	-	-
Ciconia maguari	FJ027377.1	KAARG268-07	MN356211.1
	JQ174456.1	USNMA390-10	DQ485896.1
	JQ174457.1	USNMA391-10	U72772.1
	JQ174458.1	USNMK269-11	-
	JQ174455.1	USNMK270-11	-
	MN356211.1	-	-
	-	-	-
	-	-	-
Ciconia nigra	NC_023946.1	GBMTG4880-16	KY767670.1
	KF906246.1	GBMNA11115-19	KF906246.1
	AY660580.1	GBIR7379-19	NC_023946.1
	AY567882.1	GBIR1244-09	MK818509.1
	KY767670.1	GBIR10477-19	U72771.1
	MK818509.1	-	AY567910.1
Ciconia stormi	-	-	U72776.1
Ephippiorhynchus asiaticus	-	-	U72782.1
	-	-	U72780.1
Ephippiorhynchus senegalensis	-	ROMC242-07	U72781.1

	-	ROMC243-07	KX534432.1
Jabiru mycteria	JN801320.1	BOTW286-05	U72770.1
	JQ175174.1	USNMI178-11	U19611.1
Leptoptilos cruminiferus	KJ862147.1	GBMIN133498-17	U08950.1
	KJ862148.1	GBMIN133499-17	X86754.1
	KJ862149.1	GBMIN133500-17	-
Leptoptilos javanicus	-	BROMB687-07	-
	JN709936.1	-	-
	ON359972.1	-	-
Mycteria americana	DQ433031.1	BOTW290-05	AF082066.2
	DQ433030.1	BOTW293-05	DQ485895.1
	FJ027865.1	KBARG112-07	U72779.1
	JQ175426.1	USNMJ268-11	U08949.1
Mycteria cinerea	-	-	U72778.1
Mycteria ibis	MF580199.1	GBMIN134913-17	U72784.1
	KJ862150.1	GBMIN133501-17	-
	KJ862151.1	GBMIN133502-17	-
	KJ862152.1	GBMIN133503-17	-
Mycteria leucocephala	KC439344.1	GBIR5279-13	KC439298.1
	HM804932.1	-	HM804896.1
	HM804933.1	-	HM804895.1
	-	-	U72777.1
	-	-	MH645666.1
Ardea cinerea	KJ190947.1	GBMNA11545-19	KJ190947.1
	NC_025900.1	GBMTG4795-16	NC_025900.1
Eudocimus ruber	KR862292.1	GBMNA11566-19	KR862292.1
	NC_027504.1	GBMTG4604-16	NC_027504.1

No data available = -
